# Cannabinoids: New Promising Agents in the Treatment of Neurological Diseases

**DOI:** 10.3390/molecules191118781

**Published:** 2014-11-17

**Authors:** Sabrina Giacoppo, Giuseppe Mandolino, Maria Galuppo, Placido Bramanti, Emanuela Mazzon

**Affiliations:** 1 IRCCS Centro Neurolesi “Bonino-Pulejo”, Via Provinciale Palermo, contrada Casazza, 98124 Messina, Italy; 2 Consiglio per la Ricerca e la sperimentazione in Agricoltura, Centro di Ricerca per le Colture Industriali (CRA-CIN), Via di Corticella 133, 40128 Bologna, Italy

**Keywords:** *Cannabis sativa*, cannabinoids, cannabinoid receptors, neurodegenerative diseases, epilepsy

## Abstract

Nowadays, *Cannabis sativa* is considered the most extensively used narcotic. Nevertheless, this fame obscures its traditional employ in native medicine of South Africa, South America, Turkey, Egypt and in many regions of Asia as a therapeutic drug. In fact, the use of compounds containing *Cannabis* and their introduction in clinical practice is still controversial and strongly limited by unavoidable psychotropic effects. So, overcoming these adverse effects represents the main open question on the utilization of cannabinoids as new drugs for treatment of several pathologies. To date, therapeutic use of cannabinoid extracts is prescribed in patients with glaucoma, in the control of chemotherapy-related vomiting and nausea, for appetite stimulation in patients with anorexia-cachexia syndrome by HIV, and for the treatment of multiple sclerosis symptoms. Recently, researcher efforts are aimed to employ the therapeutic potentials of *Cannabis sativa* in the modulation of cannabinoid receptor activity within the central nervous system, particularly for the treatment of neurodegenerative diseases, as well as psychiatric and non-psychiatric disorders. This review evaluates the most recent available data on cannabinoids utilization in experimental and clinical studies, and highlights their beneficial effects in the prevention of the main neurological diseases and for the clinical treatment of symptoms with them correlated.

## 1. Introduction

*Cannabis* is probably one of the most ancient non-food crops cultivated by mankind; it belongs to the botanical family of *Cannabaceae*, along with *Humulus*, the cultivated hop. It is an annual, dioecious plant, though monoecious varieties have been bred, and its diploid chromosomic complement is 2*n* = 20, with 18 autosomes and a couple of sexual chromosomes (XY for male and XX for female and monoecious plants [1].

The *Cannabis* species originated from Central Asia, where it was probably domesticated over 6000 years ago, but it has since been cultivated at virtually all latitudes for a large number of end-products deriving from the seed (e.g., fatty acids and proteins), the fiber, the wooden core and from the inflorescences, where cannabinoids are produced and secreted [2]. There still is limited agreement on whether *Cannabis sativa* should be considered a single species or a poly-species genus; however, the species boundaries, if existing, are weak, as full intercrossing between the different *Cannabis* accessions can occur, and several molecular markers-based analyses confirmed that *Cannabis* is a highly heterozygous species, with the intra-accession variation as wide as the inter-accession one [3].

In recent years, the debate on *Cannabis* re-introduction in our agricultural landscapes went beyond the agronomical and productive virtues of the plant, and especially focused on the potential of the plant’s main metabolites, the cannabinoids, as medicines useful for a number of therapeutical applications [4].

In fact, medications based on *Cannabis* have been used for therapeutic purposes in many cultures for centuries [5], with descriptions of its effects including alterations in mood, cognitive functions, memory and perception of the user [6].

In Europe, they were used at the end of the 19th century to alleviate a wide variety of conditions, including pain, spasms, dysentery, depression, sleep disturbance and loss of appetite [7]. In the first half of the 20th century cannabinoid medications fell into almost complete disuse, partly because scientists were unable to establish the chemical structure of the ingredients of the *Cannabis* plant (*Cannabis sativa* L.).

It was only in 1964 that the psychoactive component of the *Cannabis* resin and flowers, Δ^9^-tetrahydrocannabinol (Δ^9^-THC) was isolated [8]. Following, numerous non-psychoactive cannabinoids have been identified, such as cannabidiol (CBD), cannabigerol (CBG), cannabichromene (CBC), Δ^9^-tetrahydrocannabivarin (Δ^9^-THCV) and cannabidivarin (CBDV). These compounds exert multiple actions through mechanisms that are only partially related to modulation of the endocannabinoid system.

In recent years, a growing interest has been dedicated to the study of cannabinoids for their antioxidant, anti-inflammatory and neuroprotective effects [9,10]. Specifically, Δ^9^-THC is the most widely studied phytocannabinoid, but also the predominant psychotropic component of *Cannabis*, strongly limiting its therapeutic use as an isolated agent. Therefore, recently research focused to include non-psychotropic compounds, some of which exhibit potential as therapeutic agents in preclinical models of central nervous system (CNS) disease.

The present review focused on the current state of evidence regarding the possible usefulness of cannabinoid agents (psychotropic and non-psychotropic) in prevention of the main neurological disorders and/or in the treatment of symptoms correlated to them, at least in association with existing conventional therapy.

## 2. Current Cannabinoid-Based Drugs

Despite the illegality of *Cannabis* in most nations, a renewed interest in its medicinal properties has led to development of a number of cannabinoid-based medicines. Currently three drugs are used in clinical practice.

Dronabinol (Marinol^®^, Solvay Pharmaceuticals, Brussels, Belgium) capsules, a synthetic formulation of Δ^9^-THC, was approved by the U.S. Food and Drug Administration in 1986, for the management of nausea and vomiting associated with cancer chemotherapy in patients who have not responded to conventional antiemetic treatments [11]. Dronabinol is also used for the treatment of anorexia with weight loss in patients with HIV/AIDS [12].

Nabilone (Cesamet^®^, Valeant Pharmaceuticals International Inc, Mississauga, ON, Canada) capsules, is another synthetic derivative of Δ^9^-THC that is similar to dronabinol, but appears to be more potent. It was first approved in Canada in 1982 and is now also available in the United States and United Kingdom, still for the treatment of emesis [13].

Unlike Dronabinol and Nabilone, Sativex^®^ (GW Pharma, Ltd, Salisbury, Wiltshire, UK) is administered in an oral spray, consisting of a mixture of two extracts in approximately a 1:1 ratio (2.7 mg of Δ^9^-THC and 2.5 mg of CBD) in an alcoholic solution (50% ethanol). In Spain, Germany, Denmark as well as in Canada, United Kingdom and Italy, Sativex^®^ is used as treatment to alleviate spasticity in adult multiple sclerosis (MS) patients which did not show an appropriate response to other drugs during an initial trial period of therapy [14,15]. Compared to the oral route, its advantage is a faster plateau of plasma concentration. Also, it has been established that coadministration of CBD and Δ^9^-THC can reduce unwanted effects of Δ^9^-THC.

## 3. Synthesis and Production of Phytocannabinoids

Although *Cannabis* plant can be defined as a true “chemical factory” extremely rich in secondary compounds, therapeutic applications essentially rely on the cannabinoids. According to a recent review [16], there are almost 500 different chemical compounds synthesized by the *Cannabis* plant, and about 70 among these are cannabinoids. Cannabinoids are secondary compounds unique to the genus *Cannabis*, and therefore of taxonomic significance; they are terpenophenols, produced by the enzymatic condensation of a terpenic moiety (geranyl diphosphate) with a phenolic one (mainly olivetolic or divarinic acid).

**Figure 1:**
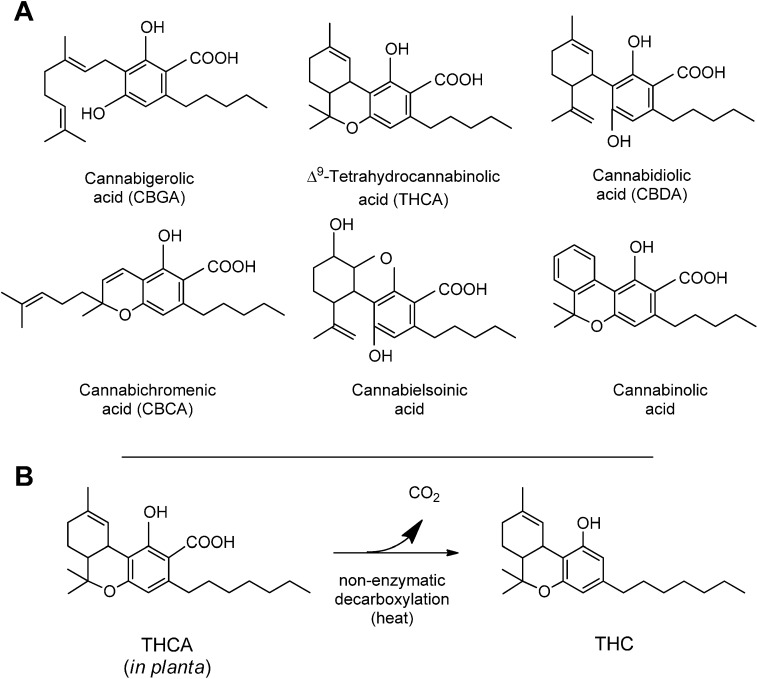
(**A**) Structure of the most common cannabinoids found in *Cannabis* plants. All the compounds have been represented in their acidic, native form, and with a pentylic side chain; (**B**) the non-enzymatic decarboxylation of Δ^9^-tetrahydrocannabinolic acid (THCA) to THC.

There are two chemical characteristics of the cannabinoid molecule that are particularly relevant (Figure 1). The first is the carboxylic group on the phenolic ring of the cannabinoid; this group is readily lost upon drying or mild heating, leaving the decarboxylated form of the different cannabinoids. It is this decarboxylation that converts the native Δ^9^-tetrahydrocannabinolic acid (THCA) into Δ^9^-THC, the cannabinoid well known for its intoxicating and psychotropic effects. All cannabinoids in *Cannabis* plants are synthesized and accumulated in their acidic form [17].

The second relevant characteristic of the cannabinoid molecule is the polyketide chain present in *meta* position to the hydroxylic group of phenolic portion (Figure 1). The most abundant cannabinoids in *Cannabis* have in this position a pentyl chain, but also propyl and even methyl side chain groups have been described [18,19].

*Cannabis sativa* accessions and varieties have been divided into chemotypes, according to the main cannabinoid they produce at maturity and to their content ratio. Five chemotypes can be recognized as most commonly occurring: chemotype I has a very low cannabidiolic acid (CBDA)/THCA content ratio, and is mainly the chemotype found in drug strains. Chemotype III, on the contrary, is characterized by a very high CBDA/THCA ratio, and is typical of all cultivated fiber varieties. Chemotype II is a mixed chemotype, containing roughly equal amounts of CBDA and THCA, as can be found in hashish strains, but also in some old fiber varieties. Chemotype IV accumulates cannabigerolic acid (CBGA) as the main cannabinoid. Finally, plants showing no cannabinoids upon gas-chromatographic analysis of mature inflorescences have been described, and for these plants the chemotype V has been proposed [20]. Clearly, plants belonging to the different chemotypes have different potentials as sources for the active principles they synthesize, and the breeding of *Cannabis* for pharmaceutical purposes had as its first target the exploration and exploitation of the variability available in *Cannabis* germplasm for cannabinoid synthesis.

**Figure 2:**
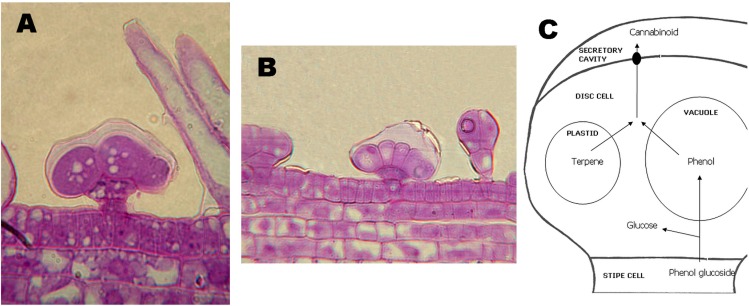
Capitate-sessile (**A**) and bulbous (**B**) glandular trichomes. In (**A**), also some non-glandular trichomes (not secerning) are visible. (**C**), schematic representation of the current model of secretion of cannabinoids from the trichomes.

The sites of biosynthesis and accumulation of cannabinoids are the glandular trichomes (Figure 2A,B). Glandular trichomes are particularly dense in inflorescences, especially on the bracts, but also the leaves and, to a minor extent, the stems of *Cannabis* plants carry trichomes. Roots and seeds are devoid of any trichome, and, accordingly, these organs contain no cannabinoids. Glandular trichomes can be capitate-stalked, capitate-sessile, or bulbous, and these different morphologies are associated with a different quantity of cannabinoids accumulated [21].

The glandular trichomes density is a trait especially important when breeding *Cannabis* for pharmaceutical purposes. In nature, the meaning for the plant’s fitness of the accumulation of cannabinoids in trichomes is still debated; it has been proposed that the conjugate bonds system characterizing THCA might have helped to protect plant functions from UV, a hypothesis partially supported by the origin of high-THCA *Cannabis* strains in regions with a high UV irradiance.

**Figure 3:**
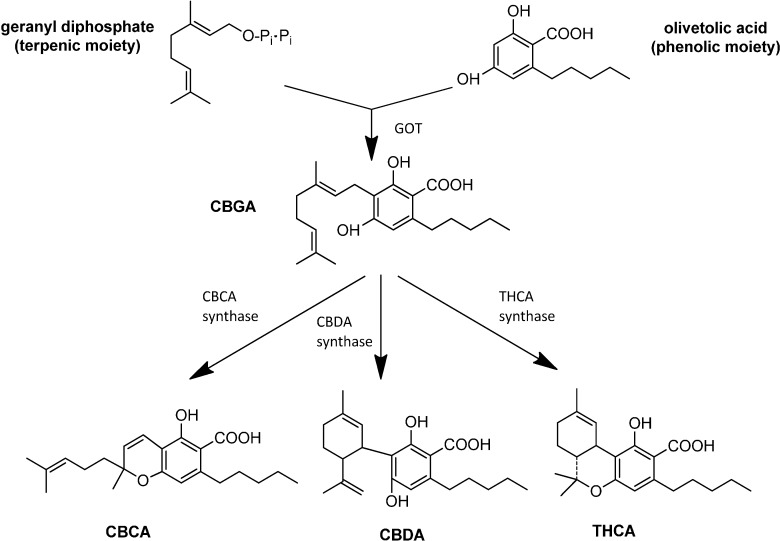
The biosynthesis of the main cannabinoids.

The first committed step in the biosynthesis of cannabinoids is the prenylation of terpene geranyl diphosphate with olivetolic acid (or, less frequently, divarinic acid), to yield the cannabinoid considered today to be the precursor of all other cannabinoids, the CBGA (Figure 3). This enzymatic step is catalyzed by the enzyme geranylpyrophosphate: olivetolate geranyltransferase (GOT). The length of the side chain (determined by the preferential use of olivetolic or divarinic acid as the phenolic component of the cannabinoid) is a genetically determined trait, though specific genes involved have not yet been identified [22]. From the pharmaceutical point of view, this “variations on the theme” due to the different length of the alkylic side chain has a great potential, as it is likely that each member of the alkyl-homologs series for each cannabinoid could be endowed with different and specific therapeutical properties [23].

CBGA is the precursor of the most abundant cannabinoids deriving from enzymatic transformation, *i.e*., THCA, CBDA and cannabichromenic acid (CBCA). These three cannabinoids are synthesized through the oxidocyclization of CBGA mediated by three specific enzymes, THCA-synthase (THCAs), CBDA-synthase (CBDAs) and CBCA-synthase (CBCAs) (Figure 3). These enzymes have been isolated from inflorescences of different *Cannabis* strains or growth stages, and biochemically characterized in detail [24].

The hypothesis that the THCAs and CBDAs genes were alleles at the same locus, and that therefore the two proteins were isoenzymes, found confirmation by in-depth genetic analysis. The cross of pure-THCA breeding lines with pure-CBDA ones systematically yields F1 progenies producing equal amounts of both cannabinoids; besides, upon selfing or intercrossing of F1 plants, the F2 offspring obtained showed a perfect 1:2:1 segregation of pure-THC: mixed THC+CBD: pure CBD chemotypes [19], as expected for a single locus (termed *B*) with two codominant alleles, *B*_T_ and *B*_D_, respectively, coding for THCAS and CBDAS. These data confirmed that, despite the several environmental factors able to modulate the total amount of cannabinoids, the chemotype (*i.e*., the THCA/CBDA content ratio) has a simple Mendelian inheritance, while the amount of cannabinoids produced by the plant is a typically quantitative trait. The two aspects of inheritance of cannabinoids had a great impact on breeding of *Cannabis*, mainly for pharmaceutical purposes.

Nowadays, the increased availability of sequence data for several *Cannabis sativa* strains related to genes encoding for the biosynthesis of secondary compounds of therapeutic interest has led to the development of advanced tools for breeding and selection of therapeutic *Cannabis* varieties. For their use in modern pharmaceutical industry, these varieties are highly uniform, and devoted to the production of a specific single cannabinoid, or of a specific blend of different cannabinoids; even the zero-cannabinoid varieties have been used in clinical tests as *placebo*. The completion of the sequencing of the *Cannabis* genome and the extensive characterization of the alleles encoding for different cannabinoid synthase variants, promises to further widen the portfolio of phytocannabinoids available for therapeutic applications; besides, the recent definition of the tertiary structure of THCAS by X-ray crystallography at the 2.75 Å resolution, with the identification of specific aminoacids crucial for enzyme function, pave the way for several biotechnological applications for synthesis of the cannabinoids *ex planta* [25].

## 4. Cannabinoid Receptors

In the human body there are specific binding sites for cannabinoids, distributed on the surface of many different cells. To date, two types of receptors have been identified to have different tissue distribution and mechanisms of signaling.

CB1 receptors, of which CB_1A_ and CB_1B_ represent two subtypes [26,27], are localized in the CNS [28]. Particularly, in the brain CB1 receptors are mainly expressed in areas involved in motor coordination and movement (cerebellum, basal ganglia and *substantia nigra*), attention and complex cognitive functions (cerebral cortex), learning, memory and emotions (amygdala and hippocampus) [29,30].In addition, CB1 receptors are present to a lesser extent in some organs and peripheral tissues, including endocrine glands, leukocytes, spleen, heart and part of the reproductive, urinary and gastrointestinal systems [31].

CB1 receptors reduce neuronal cell activity and interfere with the release of some neurotransmitters, such as serotonin, gamma-aminobutyric acid (GABA), acetylcholine, dopamine, histamine, glutamate and noradrenaline, preserving the CNS from overstimulation or over-inhibition that may be caused by other neurotransmitters.

CB2 receptors are expressed predominantly in cells of the immune system [31] and hematopoietic, but more recently their presence has been detected in the brain, in particular microglial cells, though at low concentrations [32]. It is well known that in response to damaging events, such as neuro-inflammation and cerebral hypoxia-ischemia, microglial cells may upregulate CB2 receptors expression in brain. Indeed, CB2 receptors exhibit potent anti-inflammatory effects modulating the release of cytokines [33,34].

Both CB1 and CB2 receptors belong to the family of G-protein coupled receptors (GPCRs) that, after cannabinoid agonist binding and signaling, exert an inhibitory effect on adenylate cyclase activity [35,36]. This inhibits the conversion to cyclic adenosine triphosphate (ATP) to cAMP, an important cellular secondary messenger involved in the mechanisms of signal transduction, which activates kinase protein A (PKA).

CB1 and CB2 receptors signaling leads to the downstream activation of all mitogen-activated protein kinase (MAPK), p44/42, p38 and c-JUN amino terminal kinase, which can regulate nuclear transcription factors. Also, their activation is strictly linked to ion channel regulation by inhibition of calcium channels and activation of potassium channels [37].

There is increasing evidence supporting the existence of additional cannabinoid receptors (no-CB1 and no-CB2) in both central and peripheral system, identified in CB1 and CB2- knockout mice [38,39]. Indeed, some actions of certain cannabinoid ligands seems that are mediated by other receptors like transient receptor potential vanilloid type 1 (TRPV1), G protein-coupled receptor 55 (GPR55), G protein-coupled receptor 18 (GPR18), G protein-coupled receptor 119 (GPR119) and 5-hydroxytryptamine receptor subtype 1A (5-HT1A).

TRPV1 is a non-selective cation channel for calcium, magnesium and sodium ions. It exhibits various activation and modulatory mechanisms, involving in the stimulation by GPCRs, noxious heat, low pH, and various endogenous cannabinoids such as anandamide (AEA), 12-hydroperoxy-eicosatetraenoic acid (12-HPETE) and N-arachidonoyl dopamine (NADA) [40]. Also, TRPV1 receptors play a role in transmission and modulation of nociception, as well as the integration of diverse painful stimuli [41]. They are found mainly in the nociceptive neurons of the peripheral nervous system, but they have also been described in CNS, specifically, in the hippocampus, cortex, and *substantia nigra* [42,43].

Orphan GPCRs, most notably GPR55, GPR18 and GPR119 have been proposed as potential novel cannabinoid receptors [44]. GPR55 is widely expressed in the brain, especially in the cerebellum. GPR55 can be characterized as a cannabinoid receptor, on the basis of sequence homology at the binding site, in fact the encoded integral membrane protein is a likely CB1 and CB2 cannabinoid receptors [45]. Also, it was demonstrated that GPR55 responds to a variety of both endogenous and exogenous cannabinoid ligands, such as Δ^9^-THC, CP55940 (CB1 and CB2 agonist), AEA and virodhamine [46] as do the cannabinoid receptors. These features led to suggest GPR55 as a putative third cannabinoid receptor [46,47]. GPR55 may be involved in several physiological and pathological processes by activating a variety of signal transduction pathways [48]. Combining with an extracellular signal and transmitting the signal across the membrane by activating an associated G-protein, promotes the exchange of GDP for GTP on the alpha subunit of a heterotrimeric G-protein complex. Also its activation promotes activation of the small G proteins rhoA, cdc42 and rac1 and a transduction mediated by the ERK1 and ERK2 cascade [49,50].

Recently a fourth potential receptor GPR18 activated by N-arachidonoylglycine (NAGly), a metabolite of AEA, has also been described [51]. GPR18 is expressed in gastrointestinal, immune and testicular tissues, as well as the striatum, cerebellum and brain stem [52]. Also, GPR18 is found on microglial cells in the brain where it regulates the migration of these cells following CNS damage or inflammation [51].

GPR119 is another orphan receptor originally identified in genome-sequencing efforts and expressed predominantly in the pancreas and gastrointestinal tract [53]. The identification of GPR119 as a putative cannabinoid receptor comes from reports of activation of GPR119 by oleoylethanolamide, a monounsaturated analogue and functional antagonist of AEA [54], although controversy remains on its physiological role.

5-HT1A receptor is a subtype of serotonin receptor expressed both as a presynaptic autoreceptor on raphè neurons, and as a major postsynaptic receptor in several brain regions including cerebral cortex, amygdala and hippocampus involved in mood, memory, emotion and stress response [55]. Activation of both pre- and postsynaptic 5-HT1a receptors decreases neuronal excitability [56,57] Also, 5-HT1A is a GPCR that inhibits adenylate cyclase and activate receptor operated potassium channel, whereas inhibits voltage gated calcium channel [58].

Particularly, it was demonstrated that CBD exerts many of its effects by binding 5-HT1A receptor. Activation of this receptor in key brain areas related to defensive responses, including the dorsal paeriaqueductal grey, bed nucleus of the stria terminalis and medial prefrontal cortex, leads to anxiolytic, antidepressant and antipsychotic effects showed by CBD [59].

Δ^9^-THC, of which are well-known psychotropic effects, is believed to perform the majority of its actions in the CNS binding CB1 and CB2 receptors [60]. Non-psychotrophic phytocannabinoids (CBD, CBG, CBC, Δ^9^-THCV and CBDV), exert multiple pharmacological effects via CB1/CB2 receptors as well as no-CB1 and no-CB2 receptors [50] involving intracellular pathways that play a key role in neuronal physiology. These compounds, especially CBD, are able to suppress the production of a wide range of pro-inflammatory cytokines, such as tumor necrosis factor (TNF)-α and interleukin (IL-)1β [61,62]. They show also a potent action in inhibiting oxidative and nitrosative stress, modulating the expression of inducibile nitric oxide synthase (iNOS) and reducing the production of reactive oxygen species (ROS) [63]. Moreover, non-psychotrophic phytocannabinoids attenuate high-glucose-induced mitochondrialsuperoxide generation and NF-κB activation, along with the expression of intercellular adhesion molecule 1 (ICAM-1) and vascular cell adhesion molecule (VCAM-1) [64]. Together, these activities suggest that these compounds can exert neuroprotective, antioxidant and anti-inflammatory effects.

**Figure 4:**
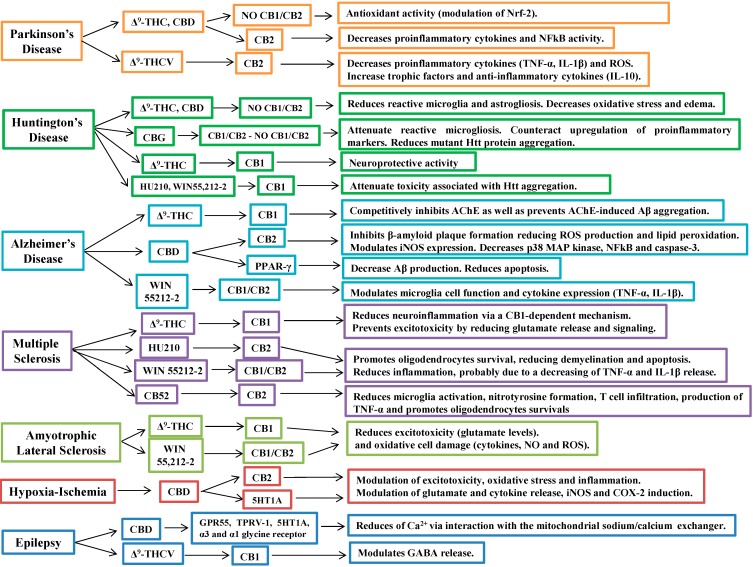
Cannabinoid-induced cellular signaling in neurologic disease.

Figure 4 summarizes the mechanisms of action and cannabinoid-induced cellular signaling in the neurological diseases investigated.

Therapeutic properties of CB receptor agonists and antagonists have been proposed for the treatment of different human disorders by preclinical and clinical observations. Interactions at CB1 and/or CB2 sites appear to affect molecular mechanisms that are responsible for disease onset or progression.

## 5. Cannabinoids in the Treatment of Neurodegenerative Diseases

Neurodegenerative diseases are chronic and progressive disorders characterized by the gradual loss of neurons in discrete areas of the CNS. Parkinson’s disease (PD), Huntington’s disease (HD), Alzheimer’s disease (AD), multiple sclerosis (MS), amyotrophic lateral sclerosis (ALS) and cerebral ischemia are considered the disorders with the highest incidence in the population worldwide.

While the etiopathogenesis of these diseases is different, a number of common mechanisms underlying their progressive nature have been elucidated, such as neuro-inflammation, oxidative stress, excitotoxicity, protein misfolding and mitochondrial dysfunction.

Nowadays, for these diseases there is no cure, current therapies have focused on treatment of symptoms and try to delay their progression. It has been demonstrated that endocannabinoid signaling is alterated in many neurodegenerative diseases [65]. Therefore, it is believed that modulation of the endocannabinoid system could be a useful alternative in neurodegeneration treatment. Furthermore, preclinical research from animal models of neurodegeneration and clinical trials have suggested a potential role of cannabinoids in the attenuation of inflammation and the protection of neurons at risk of damage. Below we reported the most significant data regarding the current status of therapeutic effects of cannabinoids in neurodegenerative disease management.

### 5.1. Cannabinoids in Parkinson’s Disease

PD is a chronic, progressive neurodegenerative disorder, characterized by the progressive degeneration of dopaminergic neurons in the *substantia nigra pars compacta* and consequent reduction in dopamine (DA) content in striatum [66]. The enzyme tyrosine hydroxylase (TH) present in all dopaminergic cells, catalyzes the formation of L-DOPA, the rate-limiting step in the biosynthesis of DA, thereby directly linking PD with TH [67]. Thus, a TH deficiency in the striatum is a hallmark of PD [68].

The death of nigral dopaminergic neurons leads to the typical motor symptoms observed in PD, bradykinesia, tremor, and rigidity.

Several experimental and clinical studies have demonstrated that endocannabinoid system undergo evident neurochemical and neurophysiological alterations after dopamine depletion [69,70,71,72]. In fact, as consequence of reduction in dopaminergic signaling, endocannabinoids levels as well as CB1 receptor expression result to be up-regulated in basal ganglia [73,74], suggesting that cannabinoids could have a therapeutic role in the treatment of movement disorders associated with PD.

To better understand what is the cannabinoid mechanism of action in PD and their antiglutamatergic effects, it is vital to explain the network of synapses involved in the genesis and in the control of voluntary and involuntary movements. For this purpose, it will be beneficial to summarize and comment on mechanism prospects outlined by many authors [75,76,77].

At level of basal ganglia, when the prefrontal sensorial cortex receives a stimulus to perform a movement, sensitive cortical neurons send glutamatergic excitatory signals to striatum nucleus (putamen) that, via GABAergic neurons, inhibits the activity of internal globus pallidus. This is known as the direct pathway of movement control. So doing, the GABAergic inhibitory signal of globus pallidus, that normally controls the activity of thalamic nucleus, is lost and thalamus can send an excitatory glutamatergic signal to motor cortex that perform the movement.

There is also an indirect pathway triggered from putamen: GABAergic neurons project to external globus pallidus that is inhibited to send, in turn, its GABAergic inhibitory signal to subthalamic nucleus. Subthalamic nucleus can now activate three pathway trough glutamatergic excitatory signals direct to: (1) *substantia nigra pars reticulate*; (2) internal segment of globus pallidus; (3) *substantia nigra pars compacta*. Among them, *substantia nigra pars compacta* is crucial to release dopamine neurotrasmitter activating striatum that stimulates the triggering of direct pathway via D1 receptors and, parallel, the inhibition of indirect pathway via D2 receptors. In PD, a depletion of dopamine in the striatum causes a cascade that lead to invert the normal balanced functioning of the basal ganglia circuitry. All this cascade of events lead to the blocking of the direct pathway and to the activation of the indirect pathway, so that we have bradykinesia as well as distorted muscle movements characteristic of PD patients.

Overall, the results is a disinhibition of the striatal neurons and therefore a relative glutamatergic overactivity, that antiglutamatergic therapies with cannabinoids counteract, mostly via CB1 receptor sited at level of presynaptic region of glutamatergic terminal [78].

More in detail, since the glutamatergic excitation is mediated by N-methyl-D-aspartate (NMDA) receptors of the neurons sited in the striatum and subthalamic nucleus, antagonists of NMDA receptors could reduce activity through the indirect pathway [77]. The result of cannabinoids action is translated in a reduction of glutamate release, decreasing calcium influx, as well as of local inflammatory events.

Current therapeutic strategies aim to increase dopaminergic transmission in basal ganglia by administration of dopamine precursors, such as L-DOPA [79], however, in a proportion of patients the efficacy of the treatment declines through time.

The majority of PD patients undergoing levodopa therapy develop disabling motor complications (dyskinesias) within 10 years of treatment. Recent studies in animal models and in the clinic propose that CB1 receptor antagonists could prove useful in the treatment of both Parkinsonian symptoms and levodopa-induced dyskinesia, whereas CB1 receptor agonists could have a role in reducing levodopa-induced dyskinesia (LID) [69].

In reserpine rat model of PD, the dopamine D2 receptor agonist quinpirole led to a significant reduction of akinesia [80]. This effect was substantially reduced by coinjection with the cannabinoid CB1/CB2 receptor agonist WIN 55,212-2. The concomitant administration of the CB1 antagonist rimonabant (SR141716A) with quinpirole and WIN 55,212-2 blocked the effect of WIN 55,212-2 on quinpirole-induced reduction of akinesia [80]. This suggests that cannabinoid antagonists might be therapeutically advantageous together with dopamine agonists in reversing the endocannabinoid effects upon inhibitory motor function observed in PD.

In Lastres-Becker *et al*. [81] study, PD was induced in rats injected stereotaxically with a 6-hydroxydopamine (6-OHDA), and then administered for two weeks with Δ^9^-THC and CBD.The authors found that both compounds were equally effective in protecting nigrostriatal dopaminergic neurons from the neurotoxin 6-OHDA. Also, it was shown that CBD can attenuate dopamine depletion and TH deficits, which are indicative of the degree of neurodegeneration of nigrostriatal dopaminergic projections [81]. These cannabinoids may function as neuroprotective agents in PD due to their capability to reduce oxidative stress. Δ^9^-THC and CBD might restore the balance between the excessive production of ROS and a relative deficiency in antioxidant properties by acting as ROS scavengers as well as improving antioxidant enzymes through the activation of signaling triggered by nuclear factor-erythroid 2 (Nfr-2) [82].

Also, CBD showed anti-inflammatory properties, reducing the generation of pro-inflammatory cytokines, such as TNF-α and IL-1β, as well as ROS and anti-inflammatory cytokines like IL-10 [83].

Moreover, Δ^9^-THCV has been shown to have neuroprotective effects, both in rats subjected to injection of 6-OHDA [84] as well as in mice injected with lipopolysaccharide (LPS) [84], possibly mediated through its antioxidant effects as well as through upregulation of CB2 receptors, and can therefore affect microglia activation. Also, in both models of PD, it was demonstrated that administration of Δ^9^-THCV delayed disease progression, reducing motor inhibition, presumably through changes in glutamatergic transmission [84].

However, despite the encouraging data achieved on the potential therapeutic utility of cannabinoids in PD rodent models, studies with non human 1-methyl-4phenyl-1,2,3,6-tetrahydropyridine (MPTP)-lesioned primates have also produced conflicting results.

It was been demonstrated that the therapy with plant-derived cannabinoid agonists for attenuating hypokinetic signs was useless and did not alleviate motor deficits [85,86]. In MTPT-treated common marmosets, the blockade of CB1 receptors with SR141716A (rimonabant), a cannabinoid CB1 receptor antagonist, reduced LID without affecting the anti-Parkinsonism efficacy of L-DOPA [87]. Similarly, Meschler *et al.* [88] using cynomolgus monkeys (*Macaca fascicularis*), confirmed that SR141716A did not improve motor disability. Also in the same study it was demonstrated that cannabinoid agonist levonantradol produced a decrease in locomotor activity and an increase in bradykinesia in primates. Also, cannabinoid agonists did not induce catalepsy in primates, a property that differs from their effects in rodents [88].

Furthermore, as for the animal studies, drug trials in PD patients have produced conflicting results. In a randomized, double-blind, placebo-controlled study the cannabinoid receptor agonist significantly reduced LID in PD [89]. On the contrary, in a double-blind, cross-over study, *Cannabis* extracts, while well tolerated, did not show effects on LID [90].

### 5.2. Cannabinoids in Huntington’s Disease

Huntington’s disease (HD) is an autosomal-dominant inherited disorder characterized by striatal neurodegeneration. Literature reports that the cause of the disease is a mutation in the huntingtin (*HTT*) gene consisting of a CAG triplet repeat expansion translated into an abnormal polyglutamine (polyQ) tract in the amino-terminal portion of huntingtin (Htt) protein [91]. Htt aggregation and its accumulation are extremely toxic for striatal and cortical neuronal subpopulations [92,93,94]. The loss of motor inhibition that follows results in an evident abnormal and involuntary writhing, commonly defined as “choreiform” movements [95], associated with dementia [96] and cognitive impairment [97].

The brain region to which is ascribed the pathology is the corpus striatum that has the functional role to control both posture and gait via GABA neurons that project to globus pallidus and zona reticulata of the substantia nigra, controlling in turn subthalamic nucleus. So doing, it inhibits or gates inappropriate or uncontrolled movements [98,99].

About neuroprotective effects of cannabinoid compounds in experimental HD, three mechanism of neuroprotection have been hypothesized: CB1-dependent, CB2-dependent and CB1-/CB2-independent [96]. The first hypothesis is corroborated by the fact that CB1 receptor is early down-regulated in ongoing disease, even in asymptomatic phases, so that CB1 receptor loss could have a role in HD pathogenesis [96]. The second hypothesis born from the evidence given by CB2 receptor localization. It was observed that it is poorly expressed in striatal parenchima under healthy condition while it is progressively over-expressed during degenerative events leading to HD. In this circumstance, CB2-activation preserve striatal neurons from inflammatory insults produced by reactive microglial cells, maybe through the release of neurotrophins, anti-inflammatory cytokines and metabolic substrates [100,101].

Finally, the CB1-/CB2-independent pathway, involved in the neuroprotection during experimental models of HD seems related to some cannabinoids with antioxidant properties, such as Δ^9^-THC and CBD, since their particular phenolic structures could exert a scavenger action against ROS. Parallel, there is also the assumption of an intracellular signal regulation via the expression control of antioxidant enzymes of phase II (*i.e*., Nrf-2/ARE signaling) [96]. On this framework, there are conflicting data and the literature about it is very wide.

From a research on MEDLINE about “Huntington’s disease and cannabinoids” we got 61 results, extended to 103 when the search was related to “Huntington’s disease and cannabinoid receptor”.

Among them, noteworthy was a recent preclinical study published on PNAS on January 2014 performed on R6/2 mouse [102]. It is the most commonly used model of HD. R6/2 mouse expresses exon 1 of the human huntingtin gene with around 150 CAG repeats. It also exhibits a progressive neurological phenotype that mimics many of the features of HD, including choreiform-like movements, involuntary stereotypic movements, tremor, and epileptic seizures [103].

The paper reports that a restricted population of CB1 receptors, and more precisely those located on glutamatergic terminals, play a crucial role in the neuroprotective activity of Δ^9^-THC and, more in general, of (endo)cannabinoids, so that the authors look at these receptors as a promising target for neuroprotective strategies of therapy during HD [102].

Also, Valdeolivas *et al*., last September published in Neurotherapeutics, the neuroprotective effects of CBG treatment in two *in vivo* models of HD, such as R6/2 mutant mouse and 3-nitropropionate (3-NP) acid-lesioned mice [104].

Authors ascribe CBG effects both to cannabinoid receptor-dependent and/or independent mechanisms. So, in the toxic model of HD authors show the neuroprotective CBG capability to attenuate the reactive microgliosis and to counteract the upregulation of pro-inflammatory markers, while in genetic model of HD they describe a recovery in the deteriorated rotarod performance typical of R6/2 mice, an expression partially normalized by CBG treatment of genes linked to HD, as well as an up-regulation of BDNF, IGF-1 and PPARγ genes. Finally, CBG-treated animals showed a reduction in the aggregation of mutant Htt protein in striatal parenchyma.

Moreover, a MEDLINE research performed on “Huntington’s disease and preclinical study and cannabinoids” gave just two results: “Neuroprotective effects of phytocannabinoid-based medicines in experimental models of Huntington’s disease” published in Journal of Neuroscience Research in September 2011 [105] and the other one entitled “Sativex-like combination of phytocannabinoids is neuroprotective in malonate-lesioned rats, an inflammatory model of Huntington’s disease: role of CB1 and CB2 receptors” published on ACS Chemical Neuroscience in May 2012 [106].

The first study tests a 1:1 botanical combination of extracts enriched in either Δ^9^-THC or CBD (the main constituents of the cannabis-based drug Sativex^®^) on rats stereotaxically subjected to unilateral injection into left striatum of the complex II inhibitor malonate, inducing HD through: (1) increasing the volume of edema; (2) reducing the number of Nissl-stained cells and enhancing the number of degenerating cells (3) causing reactive microglia and astrogliosis (4) increasing oxidative stress. According to these authors, the reversion of these effects would be mediated by a CB1 and CB2 receptor-independent mechanism provided by both cannabinoids [106].

Differently by other studies, but in accordance with evidences also reported by Fernández-Ruiz *et al.* [107], the above-reported study ascribes a balanced role to CB1/CB2 receptors with a likely involvement in drug treatment showing an up-regulation of CB2 followed by a down-regulation of CB1 receptors, suggesting that CB2 receptors could play a particularly important role in the protective effect of Sativex^®^.

Furthermore, about synthetic cannabinoids HU210 and WIN 55,212-2, they seems to work in transgenic R6/1 mice, expressing exon 1 of the human HD gene carrying a 115 CAG repeat, through a mechanism mediated by G-protein alpha subtype i/o (G(i/o))-linked and ERK-dependent signal transduction [108]. This promotes the coupling of CB1 receptors to Gi/o and attenuate toxicity associated with Htt aggregation [108].

Despite the encouraging results obtained by the experimental investigations about the potential therapeutic use of cannabinoids in HD symptoms management, clinical trials have not confirmed these results. Particularly, studies performed using cannabinoids have not shown expected improvement in the hyperkinetic symptoms of HD. Consroe *et al.* [109] published one of the first clinical trials in which CBD was evaluated for symptomatic efficacy and safety in 15 neuroleptic-free patients with HD. Authors demonstrated that CBD, was neither symptomatically effective nor toxic in these patients. Also, in literature, are reported two uncontrolled, single patient studies evaluating efficacy of nabilone, but these studies yielded conflicting results for reducing chorea severity [110,111]. Thus, although nabilone induced signs of improvement in one of these studies, in the other study [110] it made symptoms worse [111]. Nabilone was also used in a double-blind, placebo controlled, cross-over study in which it induced improvements in motor and cognitive indices [112].

The data obtained recently in animal models led to suggest that the combination of different cannabinoids, such as Sativex^®^ may be an interesting tool for developing novel therapies in HD although to date there have been no results.

### 5.3. Cannabinoids in Alzheimer’s Disease

AD is the most frequently form of dementia, with an incidence of about 34 million people worldwide [113]. AD is characterized by lesions in CNS due to the formation of beta-amyloid (Aβ) plaques, neurofibrillary tangles and cortical atrophy [114,115].

It has been demonstrated that in microglia of AD patients, CB1 and CB2 receptor expression is significantly increased, while in basal ganglia and hippocampus neuronal CB1 receptor expression is decreased [116]. Therefore, endocannabinoid system might play an important role in AD pathogenesis.

To date, the majority of drugs in use for AD treatment are acetylcholine esterase (AChE) inhibitors. According to Eubanks and colleagues [117], Δ^9^-THC competitively inhibits enzyme AChE and prevents Aβ peptide aggregation in the brains of Alzheimer patients.

In rat pheocromocytoma PC12 cells and *in vivo* models, it was shown that CBD also inhibits β-amyloid plaques formation, reducing ROS production and lipid peroxidation [118].

Also, using mice inoculated with human Aβ (1–42) peptide into the right dorsal hippocampus, Esposito *et al*. [119] have demonstrated anti-inflammatory and antioxidant actions of CBD. Indeed, CBD is able to attenuate a β-amyloid plaques formation modulating iNOS expression and also decreasing p38MAP kinase and NF-κB levels. Thus, limiting propagation of neuro-inflammation and oxidative stress.

In addition, Martin-Moreno and colleagues [120] have showed that in Aβ-mice, CBD and synthetic cannabinoid WIN 55,212-2 are able to modulate microglial cell function and cytokine expression, improving learning behavior.

Also, CBD appears able to exert a beneficial effect in the amyloidogenic pathway, through a specific molecular mechanism involving peroxisome proliferator-activated receptor-γ (PPARγ) [121]. Scuderi *et al*. [121] investigated CBD as a possible modulating compound of amyloid precursor protein (APP) in transfected human neuroblastoma SHSY5Y^APP+^ cells. Achieved results indicated the CBD capacity to induce the ubiquitination of APP protein, which led to a substantial decrease in APP full length protein levels in SHSY5Y^APP+^ with the consequent decrease in Aβ production. As consequence, CBD has promoted an increased survival of SHSY5Y^APP+^ cells reducing their apoptotic rate and increasing their survival in long-term period of cell culture. All CBD effects showed were dependent on the selective activation of PPARγ [121].

In a recent paper, Aso and co-workers [122] tested the therapeutic properties of combination of Δ^9^-THC + CBD (0.75 mg/kg each) in a AβPP/PS1 transgenic mice, an experimental model of AD, which replicates the most relevant features of disease, including cognitive impairment and several pathological alterations, such as Aβ deposition, dystrophic neurites, synaptic failure, mitochondrial dysfunction, and oxidative stress damage. Authors demonstrated that mixture of the two compounds preserved memory and reduced learning impairment in AβPP/PS1 transgenic mice when chronically administered during the early symptomatic stage [122].

A significant decrease in soluble Aβ (1-42) peptide levels and a change in plaques composition were also observed in Δ^9^-THC + CBD-treated AβPP/PS1 transgenic mice, due to a reduced microgliosis and expression of several cytokines and related molecules of neuro-inflammation [122]. In this study authors suggest that combination of Δ^9^-THC + CBD exhibits a better beneficial effect than each *Cannabis* component alone and support the consideration of a *Cannabis*-based medicine as potential therapy in AD [122].

Currently, there are only limited data displaying clinical effects of phytocannabinoids on human AD. A single, open-label, non-placebo controlled study [123] performed with AD patients reported that Dronabinol derived from Δ^9^-THC has a beneficial role in reducing anorexia and improving behavior, like nocturnal motor activity and agitation.

Despite these encouraging results, the usefulness of cannabinoid based medicines for the treatment of AD awaits the results of severe clinical trials. Also, to date there are no significant data reported in the literature on the use of phytocannabinoids in the treatment of vascular dementia.

### 5.4. Cannabinoids in Multiple Sclerosis

MS is an autoimmune inflammatory neurodegenerative disease characterized by nerves demyelination in CNS [124]. However its etiology is still unknown. Therefore, in order to better understand the etiopathogenesis of MS and to find new therapeutic strategies, researchers use some experimental models. The most used is the experimental autoimmune encephalomyelitis (EAE), which mimics the main features of human MS.

Numerous studies have been performed to evaluate the role of cannabinoids on treatment of EAE-associated spasticity as well as on modulation of the neurodegenerative process.

According to a study performed using CB1-knockout mice, it was demonstrated that the mechanism of improvement spasticity was dependent on CB1 receptors, not CB2 [125]. Also, Δ^9^-THC was reported to delay or prevent signs of spasticity in EAE mice, as well as increasing survival rates and reducing neuro-inflammation via a CB1-dependent mechanism [126].

Using synthetic cannabinoid agonists of CB1 and CB2 receptors, such as dexanabinol (HU210, (–)-1,1-dimethylheptyl analog of 11-hydroxy-Δ^8^-THC) and WIN 55,212-2 in EAE mice, it was demonstrated that they promote oligodendrocytes survival via CB1 and CB2 receptor-mediated effects, potentially reducing demyelination and apoptosis [127,128]. Also, these cannabinoids were able to reduce inflammation, probably by suppression of TNF-α and IL-1β and enhances the release of anti-inflammatory cytokines such as IL-10 in brain and peripheral blood [129]. Same results were confirmed by Arevalo-Martin *et al.* [130], using Theiler’s murine encephalomyelitis virus-induced demielinating disease (TMEV-IDD) model of chronic-progressive MS. Indeed, it was demonstrated that systemic treatment with synthetic cannabinoid CB1/CB2 receptor agonist WIN 55,212-2 in TMEV-IDD mice can limit axonal loss and neuro-inflammation in animal models of MS, by modulating microglia and lymphocytre infiltration in spinal cord [130].

Also, it was demonstrated that CB52, a newly developed cannabinoid compound (AEA and Δ^9^-THC hybrid), is more effective than other commonly used cannabinoids and its protection on oligodendrocytes is mediated by the activation of the CB2 receptor [131].

Using EAE mice, Ribeiro *et al.* [131], proved that CB52 reduced microglia activation, nitrotyrosine formation, T cell infiltration, production of TNF-α, oligodendrocyte toxicity, myelin loss and axonal damage in the mouse spinal cord white matter and alleviates the clinical scores when given either before or after disease onset.

Moreover, significant alterations of the endocannabinoid system have been found in the brain of EAE and Chronic Relapsing Experimental Allergic Encephalomyelitis (CREAE) mice. Particularly, increased levels of AEA and 2-arachidonoyl glycerol (2-AG), were detected in areas associated with nerve damage in CREAE [4] and in EAE [132], when compared to non-spastic mice.

Also, reduced CB1 expression was showed during acute phases of CREAE [133] and CB2 transcription may be increased in EAE [33]. Administration of SR141716A and SR144528, CB1 and CB2 antagonists, has been shown to worsen tremor and spasticity in CREAE mice, whilst WIN 55,212-2, methanandamide and JWH-133 CB2 agonists reduced both tremor and spasticity in diseased mice [134,135]. In addition, spasticity could also be ameliorated by the inhibition of AEA reuptake and enzymatic hydrolysis, causing a subsequent increase in AEA concentration in the CNS [4].

As well-known endocannabinoids are to be released in response to a wide range of neuronal insults [136], and levels are increased in the CSF and peripheral lymphocytes of patients with MS [137]. Centonze *et al.* [137] indeed reported a relevant increase in AEA, but not 2-AG levels, in the CSF of relapsing-remitting MS patients experiencing current relapse with a strong correlation between AEA levels and the number of inflammatory lesions visible on imaging. AEA concentrations were also higher in peripheral lymphocytes of these patients; an effect associated with increased synthesis and reduced degradation of this endocannabinoid [137]. Another study also showed elevated AEA levels in MS patients when compared with healthy controls, across the clinical spectrum, this time in the plasma, again suggesting that the peripheral endocannabiboid system ma reflect those occurring centrally [138].

Benefits from cannabinoids use seen in animal studies have also been shown in the treatment of MS patients suffering spasticity, with a significant associated disability and quality of life impairment. It is clear that spasticity results from alterations in the balance, possibly secondary to selective neuronal loss, between excitatory and inhibitory neural circuits. Under physiological conditions, inhibitory signals are sent *via* the corticospinal tract to the spinal cord, but following injury, damage to the corticospinal tract, causes disinhibition of the stretch reflex, leading to reduction in the triggering threshold. This leads to loss of control of neurotransmission between muscles and CNS, resulting in uncontrolled spastic movement [139].

Current therapies for spasticity include GABA receptor agonist, baclofen, tizanidine, benzodiazepine and anxioltyics [140]. Also, local administration of botulinum toxin have also shown efficacy in clinical trials [140]. The use of phytocannabinoids may be useful in MS patients, which show resistance to these conventional therapies, as shown in clinical studies reported in literature.

The Cannabinoids in MS (CAMS) study [141], a double-blind, randomized, placebo-controlled trial, was the first large-scale study designed to test the hypothesis that cannabinoids may have a beneficial effect on spasticity associated with MS. This study involved 630 MS patients treated with dronabinol (a synthetic Δ^9^-THC), cannador (2.5 mg of Δ^9^-THC, 1.25 mg of CBD, and 5% of elements other than cannabinoids per capsule) and placebo. It did not show any significant improvement in spasticity at 15 weeks [141], but this was evinced with both *Cannabis* compounds after one year of treatment [142]. Also, MS patients perceived a significant improvement in pain and sleep disorders [142]. Other studies performed with smaller numbers of patients and crossover studies [14] have confirmed the same results previously obtained.

Following CAMS study, comes the Cannabinoids Use in Progressive Inflammatory brain Disease (CUPID) study [143], another double-blind, randomized, placebo-controlled trial (duration of three years) in United Kingdom involving 493 patients with progressive MS. The full results from this study are pending, but initial data shows that dronabinol has no overall effect on MS progression, measured with the Expanded Disability Status Scale (EDSS) scale.

Analysis of a subgroup of patients in this study suggested a possible benefit from dronabinol in those who began the trial with milder disability, but not in those who began the trial with more severe disability [143].

A recent randomized, double-blind, placebo-controlled study involving 15 relapsing-remitting MS patients with MS-induced neurophatic pain was conducted to evaluate Nabilone combined with gabapentin. Results suggest that Nabilone as an adjunctive to gabapentin is an effective, well-tolerated combination for MS-induced neurophatic pain and thus can be used as a novel therapeutic combination in MS treatment [144].

In addition, use of Sativex^®^ has been extensively investigated in the management of patients with MS [14,15]. Currently, this spray preparation is used as treatment to alleviate symptoms of spasticity and neurophatic pain in adult MS patients that did not show an appropriate response to other drugs during an initial trial period of therapy. It has also been reported that Sativex^®^ shows efficacy in the treatment of bladder dysfunction, frequent in MS patients, showing a decrease of incontinence episodes and an increase in bladder retention volume. According to another study [145], MS patients treated with *Cannabis* extract; Δ^9^-THC, showed an important reduction in events of urge incontinence compared to placebo. Thus, suggesting that phytocannabinoids might compensate for the bladder neural circuitry disregulation that often accompanies disease progression in MS.

Spasticity, neuropathic pain and uncontrollable bladder and bowel are symptoms observed also in patients affected by spinal cord injury (SCI).

Therefore, use of cannabinoids and mixture of extracts could be useful in treatment of this pathology. Unfortunately, in the literature there are only a few studies that do not report interesting data.

### 5.5. Cannabinoids in Amyotrophic Lateral Sclerosis

ALS is the most prevalent form of motoneuron disease, characterized by degeneration and death of motor neuron populations in the cerebral cortex, brainstem and spinal cord [146]. Several mechanisms have been involved in ALS pathogenesis, such as neuro-inflammation, mostly mediated by excitotoxicity and oxidative damage on motor neurons [147,148].

There is rapidly emerging evidence that the cannabinoid receptor system has the potential to reduce both excitotoxic and oxidative cell damage.

Numerous studies reported in literature, have been conducted using ALS hSOD(G93A) transgenic mice, the strain predominantly used. Indeed, the disease in these animals closely mimics human ALS.

It was shown that mice treated with Δ^9^-THC exhibited an improvement of motor impairment by administration of the molecule, either before or after signs onset, a prolonged survival by 5% [149]. According to Bilsland *et al.* [150], a significant delay was found in disease progression when CB1/CB2 receptor agonist WIN 55,212-2 was administered to ALS hSOD(G93A) mice beginning after onset of motor impairment and tremor (at 90 days old), however, survival was not extended.

Furthermore, using the same experimental model of ALS, it was demonstrated that CB1 deletion, had no effects on disease onset, but extend lifespan by 15 days, a 13% increase in survival [150].

Also, it is important determining CB2 receptor role, since microglia from ALS hSOD(G93A) mice seems to possess increased cytotoxic potential [151]. Indeed, CB2 activation blocks β-amyloid induced microglia activation [152]. On the contrary, with other stimuli, CB2 activation showed increasing microglial migration and proliferation.

Using selective CB2 agonist, AM1241, it was reported that ALS hSOD(G93A) mice showed slowing of disease progression if administered after disease onset [153]. Administration at the onset of tremors delayed motor impairment in treated mice when compared with vehicle controls. Also, in these mice an increase of 56% in survival interval was shown [153].

In a recent study, Moreno-Martet *et al.* [154] evaluated neuroprotective effects of Sativex^®^ in SOD(G93A) transgenic mice. Sativex^®^ has proven to be effective in delaying ALS progression in the early stages of disease and in animal survival, although the efficacy was decreased during progression of disease. Also, it has been demonstrated that changes occur in endocannabinoid signaling, particularly a marked up-regulation of CB2 receptors in SOD(G93A) transgenic mice. Thus, Sativex^®^ may be used as an adjunctive therapy with only one medicine already approved, Rilutek^®^, which shows modest efficacy on disease progression.

To date, there have been few studies on human ALS. According to Yiangou *et al.* [155], in human ALS patients, spinal cord demonstrates motor neurons damages marked by CB2-positive microglia/macrophages. Moreover, a recent study analyzing activated microglia from spinal cord in human ALS patients demonstrated a CB2 increase. So all these data show how editing CB2-mediated processes could change ALS progression and how much the endocannabinoid system is potentially involved in reducing neuro-inflammation, excitotoxic, and oxidative cell damage [156].

Finally, in literature it has been reported in a single case study of patients with ALS, the 10% who admitted consuming *Cannabis*, have reported moderate relief of several symptoms, including appetite loss, depression, spasticity and drooling [157].

### 5.6. Cannabinoids in Cerebral Ischemia and Hypoxia

Ischemia is the result of a transient or permanent reduction in cerebral blood flow caused by occlusion of a cerebral artery via an embolus or local thrombosis, sufficient to alter cerebral functions. This causes a complex sequence of events, including mechanisms of excitotoxicity, release of neurotransmitters, breakdown of blood-brain barrier, inflammation, cytokines production, adhesion molecules upregulation, oxidative and nitrosative stress and programmed neuronal cell death [158,159,160].

Recently, cannabinoids have emerged as promising neuroprotective agents in several experimental model of brain damage. It seems that the endocannabinoid signaling system has various features for which appears to be involved in ischemic damage. Among these, endocannabinoids and related lipids accumulate in ischemic tissues and play a role in maintaining metabolic homeostasis and responsiveness of the brain to stress [161].

It was demonstrated that CBD can invert brain damage following cerebral ischemia in mice, decreasing brain edema and seizures associated with temporary occlusion of carotid arteries [162]. CBD was able to reduce cerebral hemodynamic impairment and ameliorate brain metabolic activity post-injury [162]. Also, it seems that CBD exerts a neuroprotective effect toward brain ischemia, causing an increase in cerebral blood flow mediated by 5-HT1A receptor and/or be secondary to its cannabinoid receptor-independent anti-inflammatory activity [163].

To date, few studies were carried out in patients with cerebral ischemia, because the limiting factor seems to be that only some compounds results are useful, and only if taken shortly before or within a few hours after cerebral damage. Clinical trials using dexabinol a synthetic Δ^9^-THC, showed no efficacy in cerebral ischemia treatment [164].

Similarly, the same mechanisms involved in cerebral ischemia, were found in hypoxic-ischemic brain injury events. Frequently, this devastating condition is one of the most important causes of neonatal brain injury and also results in adverse developmental outcomes [165].

To date, there are few reports on the possible neuroprotective effect of cannabinoids in newborns and existing publications consider their beneficial effects against excitotoxicity. CBD demonstrated neuroprotective effects in the brain of newborn Wistar rats following hypoxia-ischemia, associated with the modulation of excitotoxicity, oxidative stress and inflammation [166]. Indeed, CBD modulates glutamate and cytokines release, as well as the induction of iNOS and type 2 cyclooxygenase (COX2) [167]. Also, using a hypoxic-ischemic brain injury model in newborn pigs, Pazos *et al.* [168] confirmed that CBD modulates these mechanisms acting on CB2 and 5HT1A receptors.

Moreover, CBD activity was tested in newborn piglets, subjected to temporary occlusion of both carotid arteries plus hypoxia [162]. CBD administration reduced short-term brain damage, in a manner that can be attributed to a CBD-induced reduction of cerebral hemodynamic impairment, improvement of brain metabolic activity post-insult, reduction of brain edema, and reduction of seizures. These neuroprotective effects were not only free from side effects but also associated with some cardiac, hemodynamic, and ventilatory benefits [162].

Therefore, CBD may be considered an important candidate for future clinical trials with hypoxic newborns.

## 6. Other Therapeutic Applications of Cannabinoids

The use of *Cannabis* has been shown in the treatment of many diseases through time. Among these, treatment of epilepsy seems to be one of the most ancient.

Epilepsy is a chronic neurological disease that affects 50 million people worldwide, characterized by recurrent seizures and often accompanied by cognitive deficits and mood disorders [169]. The targeting of neuronal ion channels and both GABA and glutamate receptors has been the primary approach to eliminate convulsions. Despite the availability of a wide range of antiepileptic drugs, about one-third of individuals with epilepsy still experience seizures that do not to respond to medications [170].

The biological reason to believe that cannabinoids could suppress epileptic seizures is the abundance of CB1 receptors in some areas of the brain (hippocampus and amygdala) where partial seizures originate [171].

Various cannabinoids have been show in several clinical studies to have significant anticonvulsive properties, especially CBD and more recently CBDV and Δ^9^-THCV [172,173,174].

The antiepileptic mechanisms of CBD are not well known, since CBD has low affinity for CB1 and CB2 receptors [23], it seems that CBD may exert its effects through different mechanisms, including effects on the equilibrative nucleoside transporter, GPR55, TPRV-1, 5-HT1A, and the α3 and α1 glycine receptors. Also, antiepileptic mechanism of action of CBD might involve a reduction of Ca^2+^, via interaction with the mitochondrial Na^2+^/Ca^2+^ exchanger [175].

Likewise CBDV and, to a far smaller extent, Δ^9^-THCV produces anticonvulsant effects in animal models of epilepsy. Scutt and Williamson [176] reported that CBDV acts via CB2 cannabinoid receptor-dependent mechanisms but direct CB2 receptor effects were not shown. Recently, it was also demonstrated by other studies that CBDV acts via non-CB1/CB2 mechanisms. These compounds in fact interact with TRPV1, TRPV2, TRPA1, and TRPM8 channels, but their molecular pharmacology and mechanisms of action are less well known [177].

Additionally, CBDV has been shown to inhibit the primary synthetic enzyme of the endocannabinoid, 2-arachidonoylglycerol, diacylglycerol lipase α *in vitro* [178]. While the pharmacological significance of these effects remains unconfirmed *in vivo* and the targets identified have not yet been linked to epilepsy, they support the emergent role of multiple non-CB receptor targets [179].

Moreover, Δ^9^-THCV has demonstrated interesting potential use in treatment of convulsions. Δ^9^-THCV increases, in a GABA antagonist sensitive manner, inhibitory neurotransmission in mouse cerebellum and also exhibits anticonvulsant activity in a rat piriform cortical (PC) model of epilepsy [180]. Possible mechanisms underlying cannabinoid actions in the CNS include CB1 receptor antagonism or inverse agonism at constitutively active CB1 receptors [180].

Also, Hill *et al.* [173] have shown that Δ^9^-THCV reduced Purkinje cell firing via an increase in inhibitory neurotransmission at interneuron-Purkinje cell synapses in mouse acute parasagittal cerebellar brain slices, most likely by reducing CB1 receptor-mediated, endocannabinoid-induced inhibition of GABA release. Interestingly, Δ^9^-THCV was shown to modulate GABA release onto Purkinje cells at a network level, as it did not affect Purkinje cell spike firing following GABA-receptor blockade [181].

It is well know that CBD has therapeutic potential over a wide range of non-psychiatric and psychiatric diseases, such as anxiety, depression, bipolar disorder, psychosis and sleep disorders.

Although pharmacological effects of CBD in several biological systems have been widely investigated, mechanisms responsible for its therapeutic potential are still not clear. From studies on different animal models, it seems that CBD exerts anxiolytic-like effects by activating post-synaptic 5-HT1A receptors in key brain areas related to defensive responses, including the dorsal periaqueductal grey, bed nucleus of the stria terminalis and medial prefrontal cortex [59,182].

Other effects, such as anti-compulsive, blockade of the anxiogenic consequences of chronic unpredictable stress, increased extinction and impaired reconsolidation of aversive memories, and facilitation of adult hippocampal neurogenesis may depend on potentiation of anandamide-mediated neurotransmission. Activation of TRPV1 channels may be invoked to explain the antipsychotic effect and the bell-shaped dose-response curves commonly observed with CBD [59].

In addition to these mechanisms, CBD can interfere in different other important biological processes (inhibition of adenosine uptake, inverse agonism at CB2, CB1 antagonism, GPR55 antagonist, intracellular Ca^2+^ increase). Therefore, further studies are needed to investigate their possible involvement on CBD behavioral effects.

Russo *et al.* [183], reviewed the effects of *Cannabis*, and highlighted the benefits that can accrue in this regard, particularly with respect to symptom reduction permitting better sleep, as opposed to a mere hypnotic effect. In several clinical studies, it has been found that low doses of *Cannabis* improve mood, in particular, Δ^9^-THC increase serotonin levels in the brain, interacting with CB1 receptors.

Therefore, non-psychotropic compounds require further studies to propose these as a potentially useful drug in the treatment of a variety of intractable conditions, at least in association with current conventional therapy.

Finally, cannabinoids have been shown to be potent analgesics in animal models of hyperlgesia and thus might be useful in the treatment of inflammatory pain as well as neuropathic pain [184].

Neuropathic pain is a debilitating form of chronic pain resulting from peripheral nerve injury, toxic insults, and disease states, such as diabetes, cancer, human immunodeficiency virus, MS, and herpes zoster infection [185,186,187]. Neuropathic pain remains a significant clinical problem because it responds poorly to available therapies, needing validation of novel analgesic drugs. More recently, CBD was shown to be effective in well-established experimental models of neuropathic pain. It is believed that the analgesic effect of CBD is mediated, at least in part, by TRPV1 [188]. There is also evidence to suggest that cannabinoids can induce antinociception via supraspinal mechanisms and peripheral CB2 receptors [189]. Also, the analgesic effects may be mediated in part at the level of spinal cord via CB1 receptors activation [190].

Table 1 summarizes cannabinoid therapeutic targets for each disorder considered.

**Table 1:** Therapeutic targets for cannabinoid medicines.

Disease	Therapeutic Cannabinoids	Therapeutic Targets	Ref.
**PD**	Δ^9^-THC	Tremor	Lastres-Becker *et al.* [81]
	CBD	Dystonia and discinesia	Lastres-Becker *et al.* [81]
	WIN 55,212-2 + SR141716A (RIMONABANT)	Akinesia	Maneuf *et al.* [80]
	Δ^9^-THCV	Diskinesia	Garcia *et al.* [84]
**HD**	Δ^9^-THC	Hyperkinesia and choreic movements	Chiarlone *et al.* [102]
	CBG	Hyperkinesia	Valdeolivas *et al.* [104]
	Δ^9^-THC+ CBD (SATIVEX^®^)	Hyperkinesia and choreic movements	Sagredo *et al.* [106]
	HU210 and WIN55,212-2	Hyperkinesia	Scotter *et al.* [108]
**AD**	Δ^9^-THC	Behavior disorders and motor impairment	Eubanks *et al.* [117]
	CBD	Learning behavior	Esposito *et al.* [119]; Martin-Moreno *et al.* [120]
	WIN 55,212-2	Cognitive impairment	Martin-Moreno *et al* [120]
	Δ^9^-THC + CBD	Memory and learning impairment	Aso *et al.* [122]
	SYNTHETIC Δ^9^-THC (Dronabinol)	Nocturnal motor activity, agitation and anorexia	Walther *et al.* [123]
**MS**	Δ^9^-THC	Spasticity	Lyman *et al.* [126]
	HU210 and WIN 55,212-2	Tremor and spasticity	Molina-Holgado *et al.* [127]; Cabral *et al*. [128]; Arevalo-Martin *et al*. [130]
	JWH-133	Tremor and spasticity	Baker *et al.* [134]; Buccellato *et al.* [135]
	CB52	Motor impairment	Ribeiro *et al.* [131]
	SYNTHETIC Δ^9^-THC (NABILONE)	Neuropathic pain	Turcotte *et al.* [144]
	Δ^9^-THC+ CBD (SATIVEX^®^)	Spasticity, neuropathic pain and bladder dysfunction	Vaney *et al.* [14]; Wilkinson *et al.* [15]; Freeman *et al.* [145]
**ALS**	Δ^9^-THC	Motor impairment and spasticity	Raman *et al.* [149]
	WIN 55,212-2	Tremor and motor impairment	Bilsland *et al.* [150]
	AM1241	Tremor and motor impairment	Kim *et al.* [153]
	Δ^9^-THC + CBD (SATIVEX^®^)	Motor impairment	Moreno-Martet *et al.* [154]
**Cerebral Ischemia and Hypoxia**	CBD	Reduction of brain edema, cerebral hemodynamic impairment and seizures	Alvarez *et al.* [162]; Pazos *et al.* [166,168]
**Epilepsy**	CBD	Convulsions	Jones *et al.* [172]
	CBDV	Convulsions	Scutt *et al.* [176]; de Petrocellis *et al.* [177]
	Δ^9^-THCV	Convulsions	Dennis *et al.* [180]; Ma *et al.* [181]

## 7. Conclusions

In this review, we showed how the *Cannabis* plant, an ancient industrial crop, is drawing increasing attention as a pharmaceutical plant, and is today considered a true “bioreactor” source of botanical raw material from which high amounts of potentially valuable cannabinoids can be extracted. In the future, these molecules will be increasingly used in clinical trials necessary to assess the potential of each phytocannabinoid for the treatment of several diseases, among which CNS disorders.

Whereas, current treatments for CNS diseases are partially effective and have risks of side effects that are not easily tolerated by patients, the development of new synthetic cannabinoids or cannabinoid-derived drugs may represent an alternative strategy to pursue.

The observations from experimental models of neurological diseases, and now increasingly from clinical trials, underline the therapeutic usefulness of cannabinoids-based medicines for treatment of symptoms associated to these. In addition, there is growing evidence from experimental studies that Δ^9^-THC and other cannabinoids, notably CBD, have neuroprotective effects as a result of their antioxidant, anti-inflammatory and anticytotoxic properties which may prove disease modifying in CNS disorders.

Despite emerging evidence regarding putative therapeutic activities of cannabinoids, their effective introduction in the clinical use is still controversial and strongly limited by unavoidable psychotropic effects, exhibited by many of them.

The possibility of overcoming these side effects and developing novel approaches represents the main open question about the use of cannabinoids as new therapeutic drugs for the treatment of neurological disorders.
